# Supplementary material for: Post-Dilatation of New-Generation Self-Expandable Transcatheter Aortic Valves Does Not Increase Atrioventricular Conduction Abnormalities
Source: Diagnostics (Basel). 2023 Jan 24;13(3):427. doi: 10.3390/diagnostics13030427 (PMC9914379; doi:10.3390/diagnostics13030427)
Supplement: Supplementary file 1 [file diagnostics-13-00427-s001.zip › diagnostics-2131703-supplementary.pdf]

Supplementary Table S1. Procedural data and post-implantation outcomes in patients undergoing SE-TAVR.

	**Total (n=532)**	**No post-dilatation group (n= 428)**	**Post-dilatation group (n=104)**	**p-value**
Valve types				**0.024**
Corevalve™Evolut Pro*	115 (21.6%)	101 (23.6%)	14 (13.5%)	
Corevalve™Evolut R	417 (78.4%)	327 (76.4%)	90 (86.5%)	
Pre-dilatation	39 (7.3%)	30 (7.0%)	9 (8.6%)	0.668
General anesthesia	176 (33.1%)	151 (35.3%)	25 (24.0%)	0.029
Access sites				**0.021**
Transfemoral route	353 (66.3%)	274 (64.0%)	79 (76.0%)	
Subclavian route	179 (33.6%)	154 (36.0%)	25 (24.0%)	
Prosthesis Diameter (mm)				0.555
23	32 (6.0%)	28 (6.6%)	4 (4.0%)	
26	183 (34.4%)	143 (33.4%)	40 (38.5%)	
29	254 (47.7%)	203 (47.4%)	51 (49.0%)	
31	11 (2.1%)	10 (2.3%)	1 (1.0%)	
34	52 (9.8%)	44 (10.3%)	8 (7.7%)	
Fluoroscopy time, mean (min)	11.8±4.8	11.4±4.7	13.9±4.9	**<0.001**
Contrast medium load, median (ml)	142±38	139±35	157±45	**<0.001**
Post-procedure LVEF (%)	57.3±9.9	57.2±9.9	58.0±9.9	0.507
Post TAVR, Aortic valve area (cm^2)	1.9±0.6	1.8±0.6	2.08±0.6	0.078
Regurgitation localizations				**<0.001**
Central	25 (4.7%)	17 (4.0%)	8 (7.7%)	
Central and peri-prosthetic	7 (1.3%)	5 (1.2%)	2 (1.9%)	
peri-prosthetic *	274 (51.5%)	204 (47.6%)	70 (67.3%)	
Post-procedure aortic regurgitation > moderate	68 (12.8%)	38 (8.9%)	34 (32.7%)	**<0.001**
Post-procedure TAVR mean gradient (mmHg)	7.5±4.3	7.4±4.2	7.8±4.9	0.535
Length of hospital stay (Days)	8.3±4.7	8.3±4.7	8.4±4.7	0.823

LVEF: left ventricular ejection fraction; TAVR: transcatheter aortic valve replacement.

Supplementary Table S2. Procedural clinical endpoints.

	Total (n=532)	No post-dilatation group (n= 428)	Post-dilatation group (n=104)	p-value
Acute kidney injury (during hospital stay)	2 (0.4%)	1 (0.2%)	1 (1.0%)	0.277
Pulmonary embolism	1 (0.2%)	1 (0.2%)	0 (0%)	0.805
Sepsis	4 (0.7%)	3 (0.7%)	1 (1.0%)	0.783
Life-threatening bleeding	3 (0.6%)	2 (0.5%)	1 (1.0%)	0.539
Minor bleeding	8 (1.5%)	6 (1.4%)	2 (2.9%)	0.285
Vascular access complications	21 (3.9%)	18 (4.2%)	3 (2.9%)	0.535
Stroke				
Per-procedure	2 (0.4%)	2 (0.5%)	0 (0%)	0.647
In-hospital	9 (1.7%)	7 (1.6%)	2 (1.9%)	0.838
Conversion to cardiac surgery	1 (0.2%)	1 (0.2%)	0 (0%)	0.622
Cardiac tamponade	1 (0.2%)	1 (0.2%)	0% (0)	0.622
Coronary obstruction during TAVR	0 (0%)	0 (0%)	0 (0%)	/
Aortic rupture	2 (0.4%)	2 (0.5%)	0 (0%)	0.827
Valvular migration during procedure	2 (0.4%)	1 (0.2%)	1 (1.0%)	0.277
Mortality				
Per-procedure	2 (0.4%)	1 (0.2%)	1 (1.0%)	0.272
In-hospital	9 (1.7%)	7 (1.6%)	2 (2.0%)	0.827
30-day	13 (2.4%)	9 (2.1%)	4 (3.8%)	0.302
1-year	49 (10.3%)	41 (10.7%)	8 (8.4%)	0.507

TAVR: transcatheter aortic valve replacement.

Supplementary Table S3. Post-implantation aortic regurgitation severity after SE-TAVR implantation.

	**Total (n=104)**	**Corevalve Evolut R (n= 90)**	**Corevalve Evolut Pro (n=14)**	**p-value**
Post-implantation regurgitation				0.532
None or mild	27 (26.0%)	24 (26.7%)	3 (21.4%)	
Moderate	54 (51.9%)	44 (48.9%)	10 (71.4%)	
Severe	23 (22.1%)	22 (24.4%)	1 (7.1%)	
Post-dilatation regurgitation				
None or mild	65 (62.5%)	54 (60.0.%)	11 (78.6%)	0.378
Moderate	36 (34.6%)	33 (36.7%)	3 (21.4%)	
Severe	3 (2.9%)	3 (3.3%)	0 (0%)	

TAVR: transcatheter aortic valve replacement.
